# Host genes and HIV infection: implications and applications.

**DOI:** 10.3201/eid0303.970323

**Published:** 1997

**Authors:** R A Kaslow

**Affiliations:** University of Alabama at Birminham, Birmingham, Alabama, USA.


### Host Genes and HIV Infection: Implications and Applications

Disease emergence often involves the introduction of a familiar microbial agent into a novel ecologic niche or the evolution of a previously unrecognized microorganism in what had ostensibly been a stable environment. So accustomed are we to emergence brought on by changes in an agent or its environment that we overlook effects of the third force of causality—the host. The easy justification for our relative indifference to the contributions of the host has been that host characteristics, especially those under genetic regulation, have less potential for rapid, epidemiologically significant evolution; moreover, the genetic mechanisms of host response have been too poorly elucidated to permit rational manipulation.

The emergence of human immunodeficiency virus (HIV), however, has been different. HIV has "emerged" so masterfully by exploiting fundamental vulnerabilities in the immune system of primates that contributions of host immunity cannot be ignored. The virus has apparently evolved from its simian cousins toward a form that is extraordinarily well adapted to humans in several ways: 1) it rapidly replicates, ensuring high mutation rates within an individual host; 2) it is readily transmissible from person to person in the absence of an animal vector; and 3) because it is not invariably lethal before the age span for most human reproduction, evolutionary pressure toward radical change, attenuation, or disappearance from the population is not strong. The enormous epidemiologic implications of these basic facts have become obvious during the decade and a half of our struggle against the virus. We cannot control it by manipulating its macroenvironment as we might a parasite carried by a vector or waterborne virus. Interrupting local transmission by setting up psychosocial or mechanical barriers has limited potential. Despite the recent highly encouraging advances in antiretroviral therapy, direct and complete pharmacologic or immunologic eradication of the virus worldwide is still an untenable prospect. So we have little choice but to search for biologic strategies that reliably interdict the host-virus relationship; to accomplish that will require insight into the fundamental mechanisms of that interaction—knowledge at the level of viral and host genetics. Indeed, modulating genetically determined features of the immune response to the virus may represent the best hope for its ultimate conquest. Recent breakthroughs have accelerated the accumulation of the knowledge necessary to accomplish that aim. In this issue of Emerging Infectious Diseases, the review of current information by McNicholl and colleagues about the genetics of virus-host interaction concentrates on the recently described variations in genes encoding the human β-chemokine receptors, appropriately providing perspectives from both laboratory and public health sciences.

The quest to identify immunogenetic determinants of the host-virus interaction in HIV infection actually began with studies of the human major histocompatibility complex (HLA) soon after the AIDS epidemic was recognized, but in the past 2 years molecular technology has been focused on promising loci in the chemokine receptor gene systems, as well as in HLA. The importance of polymorphic variants of these host genes in determining whether the infection occurs and how rapidly it proceeds has been established.

The extreme polymorphism and other related properties of HLA have made it more difficult than expected to demonstrate the full influence of products of these genes on the initiation and progression of HIV infection; however, current work on HLA is slowly confirming that expectation, which is reasonably based on 25 years of research on the role of antigen-presenting genes in a whole range of autoimmune, inflammatory, and infectious processes. In contrast, β-chemokines and the genetically mediated variation in their receptors were recognized only recently, but the initial observations and numerous confirmatory reports of their involvement in HIV infection have been compelling, and there is undoubtedly more to come.

The most important consequence of these recent discoveries has been to foster an aggressive academic and industrial enterprise aimed at developing a safe, clinically beneficial immunomodulation of β-chemokines and their receptors in both infected and uninfected persons. The relative simplicity of the gene system, the frequency of the apparently protective variant (i.e., the 32bp deletion) of CCR5, and the seemingly nonessential nature of either the wild or mutant form of the receptor for normal immune function have suggested that emulation of the unreceptive mutant state (e.g., by saturating the normal receptor with a specific high affinity chemokinelike antibody) might interrupt viral penetration and replication. The implication here is clear. If antibodies to the normally functioning CCR5 can block viral attachment and prevent infection of the cell most critical to propagation of the agent without collateral damage to vital host immune function, a vaccine capable of inducing those antibodies without serious adverse effects could represent an adjunct to the current antiretroviral therapeutic agents and a major breakthrough toward a primary preventive strategy not dependent on changing personal behavior. The optimism and publicity that often accompany this kind of success must be tempered with caution: the strategy depends heavily on whether HIV can circumvent this hurdle by utilizing CXCR4 or other alternative pathways of entry into cells.

However, even if the promise of preventive and therapeutic intervention based on chemokine receptor manipulation is not soon fulfilled, another tangible benefit inherent in the discovery of factors like the receptor variants and HLA polymorphisms should not be overlooked. These genetic factors, however amenable or resistant to clinical manipulation they may prove to be, have true prognostic value and therefore offer a clear, immediate opportunity to refine our ongoing evaluations of other promising therapeutic or preventive measures. Consider the randomized trial of a new chemotherapeutic agent, intentionally designed to compare its average efficacy in all trial participants with the average efficacy of the conventional agent. Because HIV-1–infected persons who are heterozygous for the CCR5-deletion progress more slowly than those who carry only the wild type, stratifying the study population according to the presence or absence of the deletion, either during randomization or during analysis, should clarify whether the benefit of the experimental regimen in study participants who also carry the more favorable genetic trait is additive or even synergistic. Moreover, in clinical settings other than randomized trials, the additional information about receptor deletion status may be essential to analyzing the effects of interventions under evaluation or to customizing patient care.

The possibility that the genotype information might be used to refine the observations from current clinical research and to individualize the management of HIV-infected or even uninfected persons has also raised questions about whether typing more routinely might be appropriate. Although the concept of identifying a predisposing factor and modifying recommendations for treatment or prophylaxis accordingly is well established in the management of infectious diseases, screening for a particular genetic trait is not. So another implication of the research on host genetics in HIV infection is that it will probably draw health professionals into many of the same opportunities, obligations, and ultimately controversies that already surround the discovery of genes predisposing to cancer or chronic metabolic diseases like hemochromatosis. What may distinguish genetic screening in the context of infectious diseases from the rest, and even impose greater urgency for decisions about genetic testing, is that carriers of a genetic trait conferring relatively high risk may be readily capable of taking explicit precautions to avoid exposure to an identifiable etiologic agent. In short, in some situations the payoff may be more immediate.

The discovery of host genes that exert major influence on the acquisition and progression of HIV infection has radically altered our thinking about the pathogenesis of retroviral infection. The prognostic value of these genetic factors should be incorporated into the assessment of interventions to control the infection. The intense effort under way to translate knowledge of these human genetic traits into clinical benefit for HIV-infected and uninfected persons reflects a new rationale for research on emerging infectious diseases: consider the host, as well as the agent and the environment.

**Richard A. Kaslow**
University of Alabama at Birmingham
Birmingham, Alabama, USA

